# Understanding the treatment of acute pancreatitis and its complications -a database for assessing traditional Chinese medicine use

**DOI:** 10.3389/fphar.2025.1583040

**Published:** 2025-06-20

**Authors:** Xin Zhou, Yu Yang, Ya-Li Liu, Wei-An Hao, Xin-Yi Ao, Jian-Qin Liu, Yang Zhang, Zhi Li

**Affiliations:** ^1^ Department of Spleen and Stomach Diseases, The Affiliated Traditional Chinese Medicine Hospital of Southwest Medical University, Luzhou, Sichuan, China; ^2^ The Key Laboratory of Integrated Traditional Chinese and Western Medicine for Prevention and Treatment of Digestive System Diseases of Luzhou City, Affiliated Traditional Medicine Hospital of Southwest Medical University, Luzhou, China; ^3^ School of Healthcare Technology, Chengdu Neusoft University, Chengdu, China; ^4^ Innovative Institute of Chinese Medicine and Pharmacy, Academy for Interdisciplinary, Chengdu University of Traditional Chinese Medicine, Chengdu, China; ^5^ School of Integrated Traditional Chinese and Western Clinical Medicine, North Sichuan Medical College, Nanchong, China

**Keywords:** acute pancreatitis, herbal medicine, database, similarity analysis, potential effects

## Abstract

**Introduction:**

Integrative medicine combining traditional Chinese medicine (TCM) with biomedicine has become a notable approach for treating acute pancreatitis (AP). However, the absence of a comprehensive and reliable database to store and organize TCM-related data for the prevention and treatment of AP presents a significant challenge for the development of herbal medicines. To develop a comprehensive, user-friendly platform for browsing, querying, and analyzing TCM-related data for treating AP.

**Methods:**

TCM-related data for treating AP were systematically extracted from the literature and established databases. The front-end interface was developed using HyperText Markup Language, Cascading Style Sheets, and JavaScript to enhance user experience. The back-end employed Hypertext Preprocessor and My Structured Query Language for improved performance and security. The Smarty template engine was utilized to separate the front-end and back-end, facilitating efficient updates to the TCMAP platform.

**Results and discussion:**

The current version of the TCMAP includes: (i) documentation of 200 evidence-based TCM formulations, with comprehensive details on clinical applications, targets related to AP, and target pathway enrichment analysis, including 449 herbs; (ii) records of 58 natural metabolites, involving extensive information on their potential targets and pharmacological properties; and (iii) integration with a web server enables users to conduct similarity and enrichment analyses of input prescriptions based on herbs, metabolites, and genes. Furthermore, TCMAP also supports discovering anti-AP herb pairs with similar metabolites and targets. Network analysis further provides users with the intrinsic properties of anti-AP formulations. The TCMAP provides a robust data platform for predicting new metabolites and exploring the potential mechanisms of TCM in treating AP. The TCMAP is accessible at https://cellknowledge.com.cn/tcmap.

## Introduction

Acute pancreatitis (AP) is primarily characterized by localized inflammation of the pancreas, which may be accompanied by dysfunction of other organs. Integrative Chinese and biomedicine is a significant approach for treating AP in China. Integrative Medicine is a medical model that systematically combines traditional medicine with modern medical theories and practices, aiming to enhance treatment efficacy by leveraging complementary strengths (Dossett et al., 2017; Korzenik et al., 2017). In the treatment of AP, Integrative Medicine is typically manifested as follows: In terms of diagnosis, biomedicine determines the severity of AP through imaging (e.g., CT) and biochemical indicators (e.g., serum amylase), while TCM guides the selection of prescriptions through syndrome differentiation (e.g., damp-heat in the liver and gallbladder syndrome, excessive heat and constipation in the interior syndrome). Regarding treatment, biomedicine adopts essential therapies such as fluid resuscitation and antibiotics, while TCM uses prescriptions (e.g., Dachengqi Decoction) to regulate intestinal function and suppress inflammatory responses. Clinical studies have shown that the combined treatment of Western and Chinese medicine can significantly shorten the hospital stay of AP patients (Miao et al., 2018; Cai et al., 2025). Among these treatments, herbal formulas have shown significant advantages in targeting multiple pathways to prevent and manage AP and its related complications, with their efficacy recognized in many Asian countries (Jiang et al., 2020). In 2021, the “*Guidelines for Diagnosis and Treatment of Acute Pancreatitis in China (2021)”* recommended traditional Chinese medicine (TCM), including *rhubarb*, *Trii Sulfas*, and various formulations such as Qingyi Decoction and Dachengqi Decoction, as effective adjunctive strategies for promoting gastrointestinal recovery in AP patients. A meta-analysis of 23 randomized controlled trials involving 1,865 AP patients found that Chengqi-series decoctions significantly improved clinical outcomes, including relief of abdominal pain, reduction of multiple organ dysfunction syndrome (MODS), and decreased mortality rates (Lin et al., 2023). Recent studies have found that combining TCM with biomedicine was more effective in reducing AP patients’ hospital stays than biomedicine alone (Wan et al., 2012; He et al., 2022; Guo et al., 2023; Li et al., 2023). Therefore, integrating TCM in preventing and treating AP and its complications holds significant promise.

**FIGURE 1:**
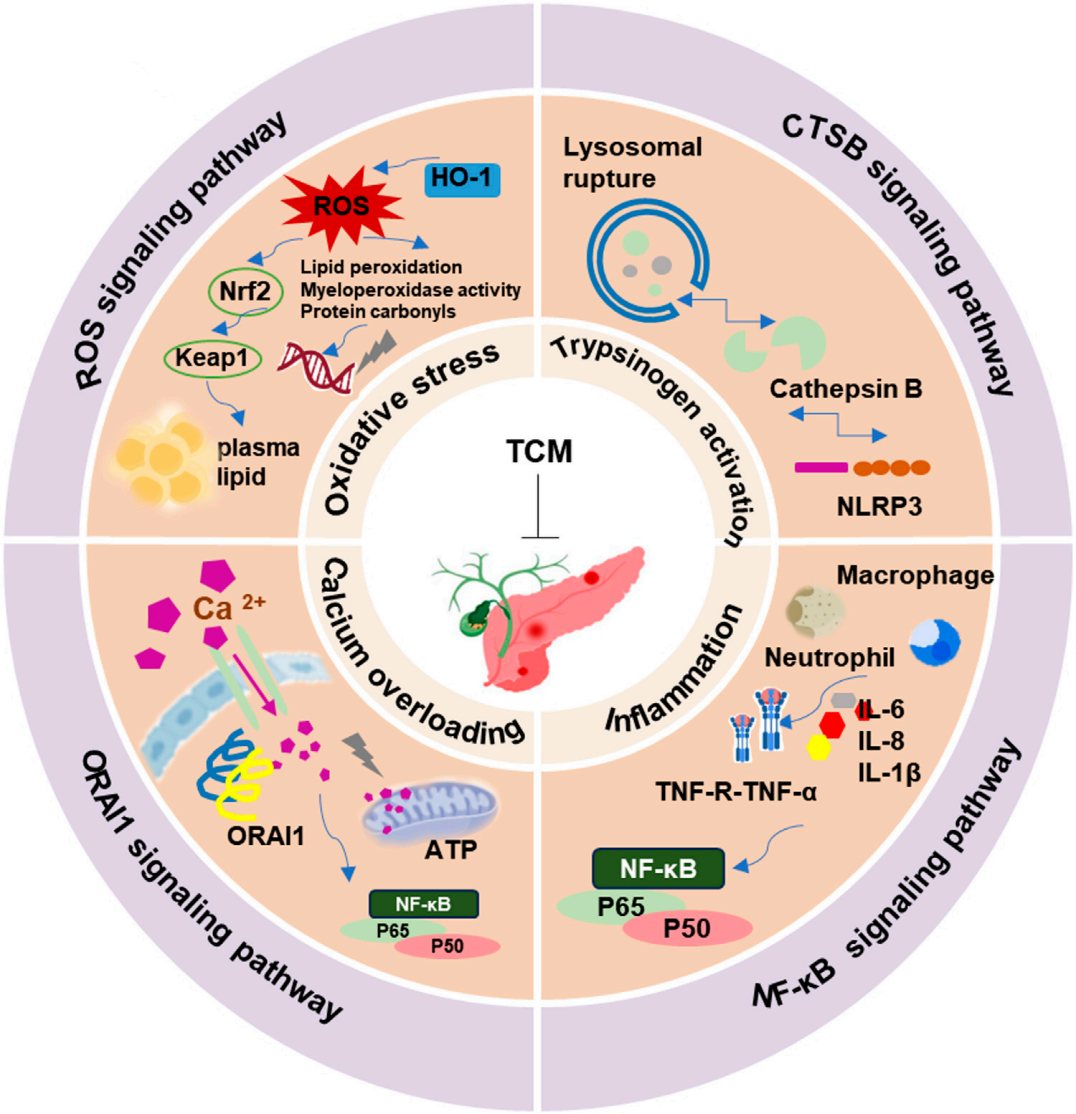
The potential therapeutic effect of TCM on AP. Natural medicines can ameliorate AP by inhibiting abnormal trypsin activation, calcium overload, inflammatory responses, and oxidative stress. These effects are mediated by regulating CTSB, ORAI1, NF-κB, and ROS signaling pathways.

Research into the potential mechanisms by which TCM improves AP has also advanced. Advancements in high-throughput experimental technologies have significantly enhanced research on the pharmacological effects and likely targets of TCM in improving AP. Between 2007 and 2023, 756 authors from 147 institutions across 13 countries published studies on TCM for AP in 76 academic journals (Lan et al., 2024; Yang et al., 2024). These advanced studies have identified critical targets of TCM in the prevention and treatment of AP, including lysosomal cathepsin B (CTSB), calcium channel protein (ORAI1), nuclear factor-κB(NF-κB), and reactive oxygen species (ROS) (Hu et al., 2023; Ishqi et al., 2023). These targets are implicated in critical biological processes such as abnormal trypsin activation, calcium overload, inflammatory response, and oxidative stress (Figure 1). Moreover, multi-omics technologies are widely employed to identify critical natural active metabolites of TCM and to characterize metabolic and transcriptomic changes following intervention in AP disease models. Qingyi Decoction, a classic TCM formula, has demonstrated significant efficacy in treating AP and its complications. Liquid chromatography-tandem mass spectrometry (LC-MS/MS) has revealed that Qingyi Decoction leads to metabolites such as rhein, baicalin, and paeonol in the circulatory system and tissues. Notably, tetrahydropalmatine and corydaline accumulate significantly in the pancreas, elucidating the positive regulatory effects of Qingyi Decoction on AP-induced dysbiosis and gastrointestinal dysfunction (Ma et al., 2024). Recent studies have found that Urolithin A (UA), a natural metabolite produced from the metabolism of Ellagic acid (EA) by gut microbiota, can mitigate severe acute pancreatitis (SAP) by modulating the ER-mitochondrial calcium channel and reducing necrotic apoptosis in acinar cells (Kang et al., 2024). A substantial body of research evidence has been accumulated regarding the use of TCM for treating AP and its related complications. However, the relevant data remain dispersed and lack systematic organization, which hampers comprehensive mechanistic studies.

Over the past decades, researchers have utilized bioinformatics technologies to develop comprehensive TCM knowledge databases, which have been crucial in advancing the modernization of TCM research (Fan et al., 2023). With robust search, storage, and visualization capabilities, these databases are pivotal in integrating complex traditional TCM knowledge with contemporary research data. Currently, widely used comprehensive databases for TCM include TCM-Suit, HERB, TCMID, TCMSP, and SymMap (Xue et al., 2013; Ru et al., 2014; Wu et al., 2019; Fang et al., 2021; Yang et al., 2022). These databases cover a broad spectrum of information, including herbs, metabolites, targets, and phenotypes. They are extensively used for querying herbal information, drug repositioning, discovering new natural metabolites, and constructing association networks. However, these databases do not cover TCM data related to specific diseases. Moreover, specialized TCM databases have offered new insights into preventing and treating complex diseases. For example, Ren et al. developed the TCM2COVID database, a dedicated platform for recording, querying, and exploring data on TCM treatments for COVID-19 (Ren et al., 2022; Pan et al., 2025). However, the databases mentioned above do not provide specific functional modules. No comprehensive database is available that integrates the extensive and valuable experimental data and clinical evidence on TCM for anti-AP research. This resource would greatly benefit future researchers. Therefore, building on our preliminary work, we developed TCMAP, a manually curated, comprehensive database focused on anti-AP research in TCM. TCMAP provides a user-friendly, publicly accessible platform for searching, browsing, and analyzing research findings related to TCM treatments for AP.

## Materials and methods

### Data collection

We searched the PubMed, China National Knowledge Infrastructure (CNKI), and Wanfang databases for articles published between 2000 and 2023 using the keyword combination of (“acute pancreatitis” OR “AP”) AND (“traditional Chinese medicine” OR “herbal formula”) AND (“clinical trial” OR “mechanism”).

Inclusion Criteria: i. Randomized controlled trials (RCTs), cohort studies, and pharmacological mechanism studies. ii. Precise specification of the formula composition, dosage, and efficacy indicators (e.g., APACHE-II score, length of hospital stay). iii. Compliance with the clinical medication guidelines in the Clinical Guidelines for Traditional Chinese Medicine Diagnosis and Treatment and the Chinese Pharmacopoeia (2020).

Exclusion Criteria: i. Case reports, reviews, and non-Chinese or non-English literature. ii. Duplicated data or unverifiable formula information. iii. Studies beyond the clinical indications of AP or those not approved by experts in spleen-stomach diseases. Two researchers independently categorized the remaining studies (e.g., classical formulas, patented drugs). A third researcher resolved discrepancies.

All extracted data were cross-verified by two people. Two researchers independently entered the formula composition, herb names, and compound targets. Discrepancies were reviewed against the original texts. Herb names were standardized according to the Chinese Pharmacopoeia (2020) (e.g., “Dahuang” was unified as “Radix et Rhizoma Rhei”). Ten percent of the formulas (n = 20) were randomly selected, and the accuracy of the metabolite and target information was verified through the HERB database (http://herb.ac.cn/), with a consistency rate of 95%.

### Anti-AP TCM formulas

We gathered anti-AP formulas from publicly available literature databases. After eliminating redundancies, researchers manually categorized the formulas according to the “*Comprehensive Guide to Traditional Chinese Medicine Formulas*.” In the TCMAP database, these formulas are classified into classic formulas, recommended formulas, master formulas, and Chinese patent formulas based on their sources. The “*Expert Consensus on TCM Diagnosis and Treatment of Acute Pancreatitis (2017)”* outlines TCM pattern types for AP and recommends specific formulas for each pattern to optimize therapeutic outcomes. To enhance understanding of the metabolic characteristics related to TCM formulas, TCMAP includes data on blood metabolites from 13 anti-AP formulas and will continue to update this information. Additionally, comprehensive details on these formulas—including clinical trials, application scopes, potential mechanisms, and therapeutic effects—have been sourced from the Chinese Clinical Trial Registry and original research articles.

### Anti-AP natural metabolite

In TCMAP, data on anti-AP natural metabolites were manually compiled from Peer-reviewed literature. Therapeutic information for these metabolites, including potential mechanisms, animal models, and target organs, is tabular on the data retrieval interface. Basic information on other metabolites is obtained from the PubChem database, updated in 2023 (Kim et al., 2023).

### Anti-AP herbs

In the herbal field section, we have integrated all herbs associated with the anti-AP formulas and natural metabolites discussed earlier. TCM professors standardized the names according to the “Chinese Pharmacopoeia” and “*Shenlong Bencaojing*” to address the variability in herb names across different regions and historical periods. Standardized herb names are mapped to the HERB database using Herb IDs, giving users easy access to essential information. Additionally, we have compiled a comprehensive dataset on herbal treatments relevant to AP, focusing on therapeutic classes, properties, and efficacy.

The extracted data underwent cross-validation by two independent corresponding authors to ensure consistency. We utilized MySQL for storing and managing the extracted data. A unified structured database table was created based on the anti-AP TCM formulas, herbs, and natural metabolites. Different sources of TCM data were linked through a unique identifier (TCM_ID). An Extract, Transform, Load (ETL) tool was employed for data integration, ensuring efficient retrieval and analysis within the integrated database.

### Pipeline for formula similarity analysis

For user convenience, TCMAP provides a web server for formula similarity analysis, accessible at: https://cellknowledge.com.cn/tcmap/webservice.html. TCMAP calculates the similarity between the query formula q and the recorded formula *i* using a formula similarity (FS) score based on herbs, metabolites, and targets, respectively. Herbs often represent TCM formulas’ efficacy and meridian-tropism properties against AP. Metabolites are the material basis for a formula to treat diseases. Targets reflect the biological significance of a formula in improving AP. In addition, Fisher’s exact test is then applied to assess the statistical significance of this similarity. The FS score is calculated using the Overlap similarity coefficient, where *L*_*q*_ represents the list of medicinal materials in the query formula *q,* and *L*_*i*_ represents the list of medicinal materials in the recorded formula *i* (Ren et al., 2022):

$$FS\left(q,i\right)=\frac{\left|Lq\cap Li\right|}{\mathrm{Min}\left(\left|Lq\left|,\right|Li\right|\right)}$$

### Pipeline for collaborative herb query

Based on the formula similarity analysis of metabolites and genes, we further propose mining herbs with collaborative effects against AP. The collaborative effect of herbs is the basis of formulas. TCMAP uses the metabolite similarity (MS) score to quantify the similarity between the queried herb metabolite q and the documented herb metabolite *i*. This score is calculated by the Tanimoto coefficient, where *L*_*q*_ is the list of metabolites of the queried herb metabolite *q*, and *L*_*i*_ is the list of metabolites of the documented herb metabolite *i* (Bajusz et al., 2015; Kuwahara and Gao, 2021):

$$MS_{metabolite}\left(q,i\right)=\frac{\left|L_{q}\cap L_{i}\right|}{\left|L_{q}\right|+\left|L_{i}\right|-\left|L_{q}\cap L_{i}\right|}$$

In the module for obtaining herb combinations based on gene targets, TCMAP uses the gene similarity (GS) score to quantify the similarity between the queried herb gene target q and the documented herb gene target *i*. This score is calculated by the Jaccard coefficient, where *L*_*q*_ is the gene list of the queried herb gene target *q*, and *L*_*i*_ is the gene list of the documented herb gene target *i* (Salvatore Rand et al., 2020):

$$GS_{gene}\left(q,i\right)=\frac{\left|L_{q}\cap L_{i}\right|}{\left|L_{q}\cup L_{i}\right|}$$

Finally, we used Fisher’s exact test to evaluate the significance of the similarity between the queried formulas or herbs and the documented formulas or herbs.

### Architecture of TCMAP

To optimize the storage, retrieval, and visualization functions in TCMAP, we use HyperText Markup Language (HTML), Cascading Style Sheets (CSS), and JavaScript on the front end to ensure a superior user experience. Dynamic data interaction is managed through Asynchronous JavaScript and XML (AJAX) technology to enhance usability further. The platform’s web services are hosted using Nginx. At the same time, Hypertext Preprocessor (PHP) and MySQL are employed on the back end to ensure fast query performance and data security. The Smarty template engine separates the front and back end, streamlining logic and interface content for easier future management and maintenance. To accommodate increasing data, we will deploy more MySQL database servers, utilizing clustering solutions like MySQL Cluster or load balancing technologies such as HAProxy. This approach will ensure the system’s scalability and high availability.

### Visualization of the network and enrichment analysis

To demonstrate the association between TCM formulas and the disease mechanism of AP, TCMAP provides a disease-target cross-analysis module. 6,196 disease genes are obtained from public databases (Stelzer et al., 2016; Piñero et al., 2020) and then mapped to the target genes of the formula metabolites. Finally, the intersection of the metabolite targets and AP disease genes is calculated.

TCMAP uses KEGG pathway enrichment analysis to decipher potential mechanisms of action in TCM formulas. It reads an Excel file containing the correspondence between formulas and genes (a unique TCM_ID identifies each formula). It converts the gene identifiers (Entrez ID) in each row into a list format. The enrichKEGG () function in the R language clusterProfiler package is called to evaluate the pathway enrichment significance of the target gene set based on Over-Representation Analysis (ORA) (Pomyen et al., 2015; Wu et al., 2021). Both the adjusted p-value (using the Benjamini–Hochberg method) and the q-value are less than 0.05, and the top 10 most significant pathways are retained. The dotplot () function generates a pathway enrichment dot plot, where the x-axis represents the gene ratio, the size of the dots maps to the number of genes, and the color gradient indicates the p-value.

To enable efficient dynamic visual interaction, the TCMAP platform employs a comprehensive technical architecture. First, the data loading and parsing module retrieves data from local structured TSV files, namely, the formula. tsv, herb. tsv, metabolite. tsv, and target. tsv. It extracts 11,196 gene targets mapped by metabolites from the herb database. After intersecting with AP-related disease genes, 5,000 gene targets are removed. The remaining data is then utilized to construct a multi-level relationship of formula-herb-metabolite-target, and efficient in-memory storage is accomplished via global variables. Subsequently, the interactive control initialization module leverages the Select2 plugin to enhance the user experience. It features a dropdown menu that supports fuzzy search and placeholder prompts, combined with entity-type selection buttons (for herbs, metabolites, and targets) and a submission function, thus creating an intuitive entry point for user operations. The network graph generation module serves as the core functionality. Based on the user’s selection of a formula and an entity type, it recursively explores the node dependency relationships. It dynamically generates nodes and links through the Force-Directed Graph layout of D3. js and employs SVG rendering to achieve high-fidelity visualization. In addition, the supplementary function module integrates interactive operations such as node dragging, canvas zooming, view resetting, simulation control, and SVG export (supporting vector graphics suitable for academic publications). Its core functionality relies on D3. js’s dynamic data binding (Data Join) and physical simulation engine to ensure real-time responsiveness and visual interpretability of complex networks. This technical solution strikes a balance between the robustness of data processing, the friendliness of user interaction, and the professionalism of scientific visualization. As such, it provides a reliable tool for deciphering the multi-level action mechanisms of anti-AP compound prescriptions.

## Results

### Data collection of the TCMAP database

**FIGURE 2:**
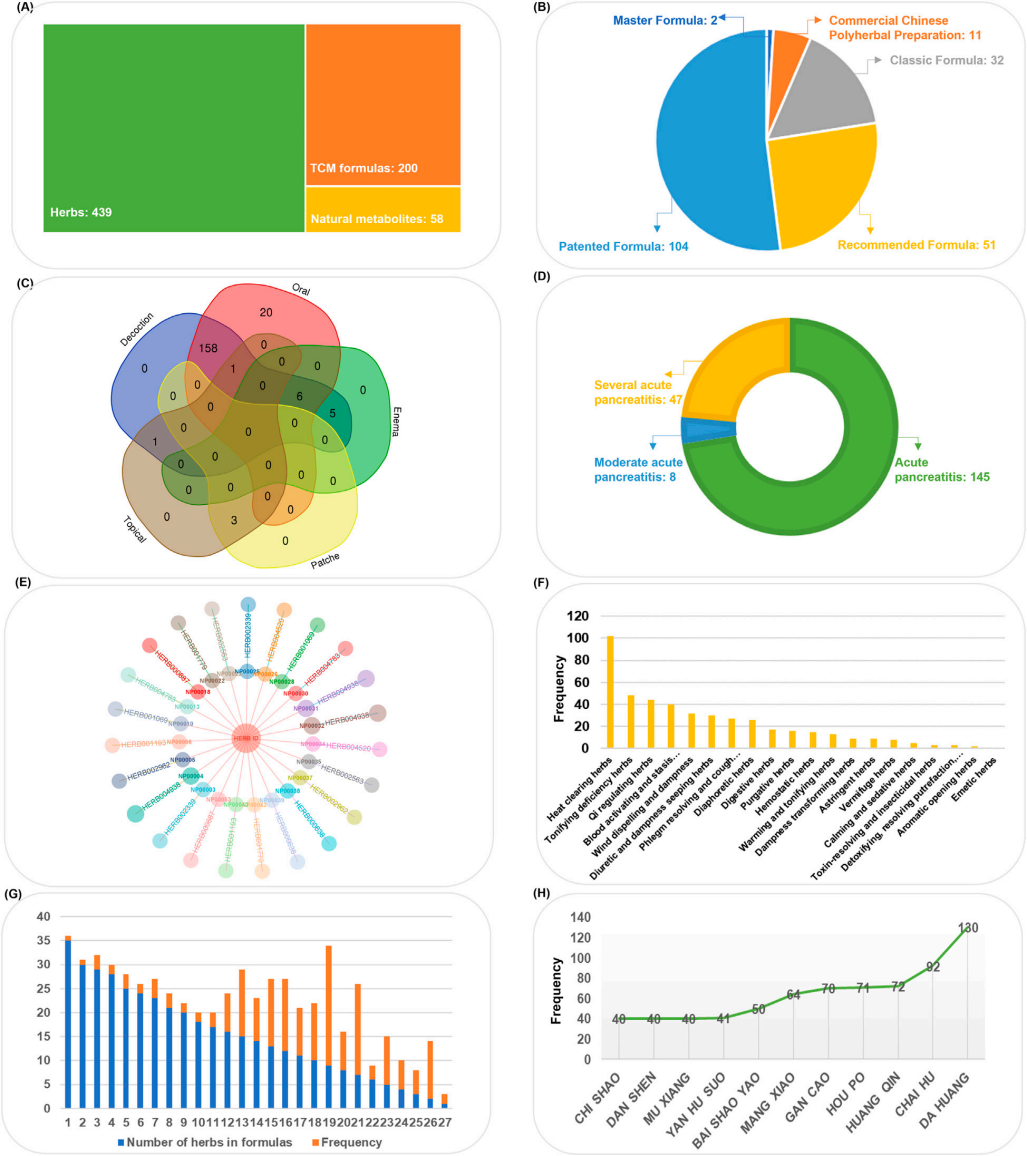
Characteristics of TCMAP data integration. **(A)** Composition of current TCMAP data. **(B)** Classification of TCM Formulas. **(C)** Typical dosage form and administration of TCM formulas. **(D)** High-frequency herbal combinations in TCM formulas. **(E)** Range of clinical application of TCM Formulas. **(F)** The frequency of herbs in formulas. **(G)** Enrichment of natural products in herbs. The inner circle represents the NP_IDs of natural products in the TCMAP database, while the outer circle uses corresponding colors to indicate the herbal sources of these natural products. **(H)** The therapeutic classes of herbs in TCMAP.

The data composition of the TCMAP database is illustrated in Figure 2. We have diligently gathered 200 anti-AP TCM formulations from existing public resources, each assigned a unique “TCMID“ (Supplementary Table S1). These formulations are categorized into Classic Formulas (32), Master Formulas (2), Patented Formulas (104), Recommended Formulas (51), and Commercial Chinese Polyherbal Preparation (CCPP) (11), and are sourced from TCM classics, modern works by renowned Chinese physicians, the Chinese patent database, clinical guidelines, and already-marketed CCPP (Figures 2A,B). These formulations typically represent hospital-specific preparations with potential for new Chinese patent development, including Chaihuang Qingyi Huoxue Fang, Zhongyao Bawei Zhitong Gao, and Qingyi Tang (Ma et al., 2024; Yang et al., 2024). The dosage forms of TCM formulations for the prevention and treatment of AP vary depending on their intended use. For example, decoctions can be administered orally and via enema, granules and powders are primarily used orally, while patches are generally applied externally to the abdomen (Figure 2C). SAP is the primary cause of increased mortality in patients with AP, but no specific treatment is available (Trikudanathan et al., 2024). Forty-seven formulas with potential benefits for improving SAP were cataloged in the TCMAP database (Figure 2D). These formulas may work by inhibiting inflammatory responses, promoting pancreatic acinar cell apoptosis, enhancing microcirculation, and regulating gut microbiota. This provides new opportunities for natural drug discovery aimed at preventing and treating SAP.

In addition to TCM formulations, natural metabolites are essential in anti-AP. To date, there are 58 studies on natural metabolites for the prevention and treatment of AP, derived from 46 different herbs, including *Common Yam Rhizome* (HERB004938), *Radix et Rhizoma Rhei* (HERB001069), (HERB000638), *Rhizoma Chuanxiong* (HERB000887), *Herba Artemisia Annuae* (HERB004520), and *Radix Glycyrrhizae* (HERB001779), et al. (Figure 2E) (Supplementary Table S2). Basic information on these natural metabolites for combating AP has been collected from the PubChem database, including molecular formulas, SMILES, and PubChem CIDs. Additionally, we have manually curated the therapeutic details of these metabolites to help users better understand the mechanisms through which they exert their anti-AP effects (Qiu et al., 2024).

The anti-AP herbal formulations and natural metabolites mentioned above involve 449 herbs (Supplementary Table S3). These herbs are classified into 20 categories based on TCM efficacy (Figure 2F). Formulas listed in the TCMAP database typically contain 9 and 15 herbs, though some formulations may include as few as one or as many as 35 herbs (Figure 2G). The herbs appearing most frequently (>50 times) in anti-AP formulations are listed in Figure 2H. Modern pharmacological research techniques have clarified the specific active metabolites of these herbal medicines that alleviate symptoms associated with AP. We obtained non-redundant metabolite information from the HERB database using each herb’s unique identifier.

### The web interface of the TCMAP database

**FIGURE 3:**
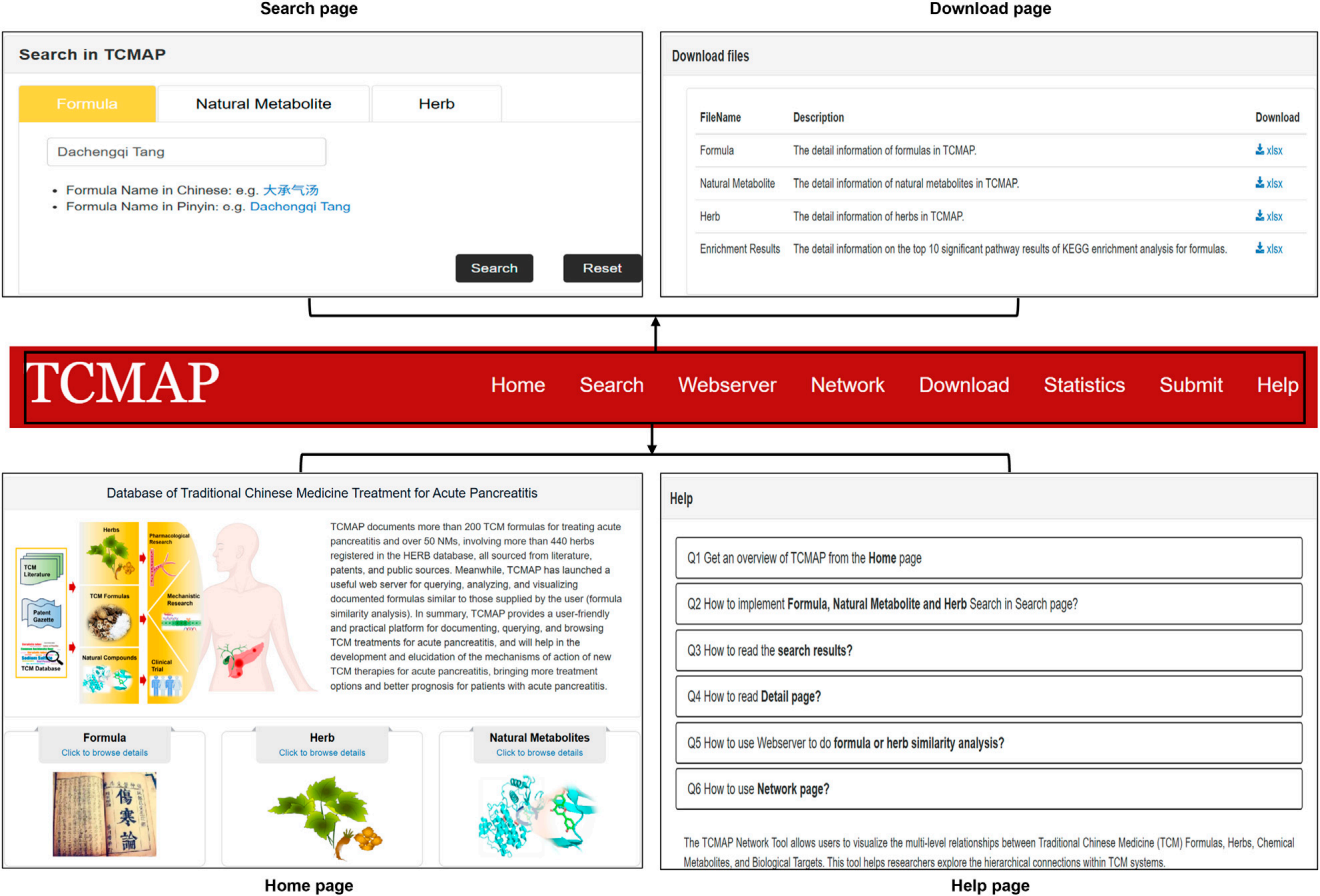
The web interface of TCMAP. Users can navigate to the Home, Search, Download, and Help pages via the navigation bar.

The TCMAP database is freely accessible at https://cellknowledge.com.cn/tcmap. It features an intuitive web interface for browsing, querying, and downloading anti-AP TCM information (Figure 3). The database’s top menu allows users to navigate effortlessly to all functional pages: Home, Search, Webserve, Network, Download, Statistics, and Help. To enhance the user experience, TCMAP adopts a fuzzy matching retrieval strategy (Brito da Silva et al., 2020). Users can enter keywords for retrieval, and the system will provide matching suggestions. Even if users fail to obtain the target data directly in the current version of TCMAP, they can still browse similar information selected through fuzzy matching. Users can explore anti-AP TCM formulations, herbal medicines, and natural metabolites on the Home page. For direct access to specific anti-AP herbs, users can search and view the basic information of herb formulations, herbs, and natural metabolites on the Search page. On the detailed interface of anti-AP formulas, TCMAP provides the targets related to AP analysis, including target name, Gene ID, and source. Meanwhile, based on the KEGG pathway enrichment results, the top 10 key pathways of the intersecting genes in AP are highlighted in the diagram (Supplementary Table S4). To accommodate user needs, all data is provided in. xls format on the Download page, accessible for free download and editing based on data analysis requirements. The Help page is a valuable resource for new users. It provides comprehensive instructions on how to use the TCMAP database. It offers standard solutions to common problems, ensuring that users can easily make the most of the platform’s features.

### Using the TCMAP database: a case study of Dachengqi Tang search

**FIGURE 4:**
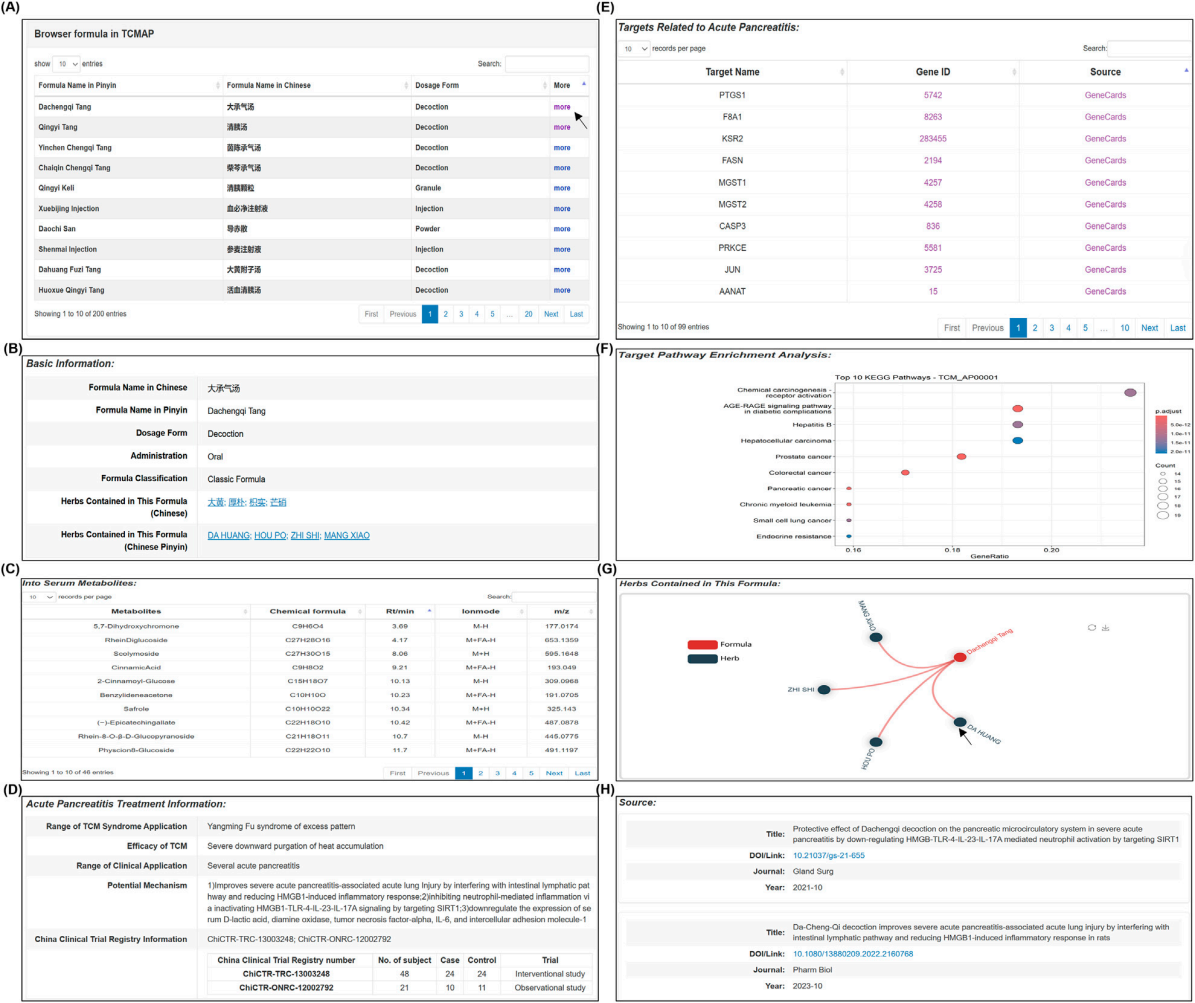
Searching and results for the Dachengqi Tang. **(A)** The TCM search page provides an overview of metabolite data. **(B–D)** The detailed module displays the latest research on retrieved formulas, including basic information, into blood metabolite details, and treatment information. **(E)** Details of the cross-analysis results of gene targets between TCM metabolite and AP disease. **(F)** Visual network diagram of target genes for anti-AP formulas. Users can download the complete dataset for metabolite information. **(G)** Herbal medicines in the formulation are displayed on the web template; hovering over a node highlights the herb and links to its detailed page. **(H)** The sources of references for the formula, along with the links to the original texts.

Dachengqi Tang, a widely studied anti-AP herbal formulation in China, is used here to illustrate utilizing the TCMAP database (Figure 4). Users can access the search screen via the navigation bar and enter the formula’s full Chinese or Pinyin name. Details of Dachengqi Tang, including Basic Information, Serum metabolites, Treatment Information, Targets Related to Acute Pancreatitis, Target Pathway Enrichment Analysis, and Source, will then be displayed in the search results. To facilitate access to information about the herbs in the formula, hyperlinks are provided in the herbal medicine names and images. Users can manually search for detailed anti-AP treatment information on specific herbs by entering their Chinese or Latin names. In the targets related to the acute pancreatitis module, users can view the intersecting genes between Dachengqi Tang and AP. Genes such as Prostaglandin-endoperoxide synthase 1 (PTGS1), Factor VIII intron 22 gene A1 (F8A1), and Kinase suppressor of Ras 2 (KSR2) indicate that the biological mechanisms by which Dachengqi Tang improves AP are related to regulating the inflammatory response, pancreatic endocrine function, and improving microcirculation disorders (Jaworek et al., 2001; Keane et al., 2011; Jeon et al., 2024). In the target pathway enrichment analysis interface, users can obtain pathway enrichment analysis results for the potential regulatory gene targets of Dachengqi Tang. In the bubble chart, the vertical axis shows the names of the pathways enriched by the formula, and the horizontal axis represents the GeneRatio. The points in different colors correspond to different significant p-values, and the size of the points indicates the number of mapped genes. Although the enrichment analysis chart only displays the top 10 pathways, TCMAP provides comprehensive pathway enrichment information in xls. Format for users to download conveniently.

### The web interface of the TCMAP webservice

#### Formula similarity analysis

**FIGURE 5:**
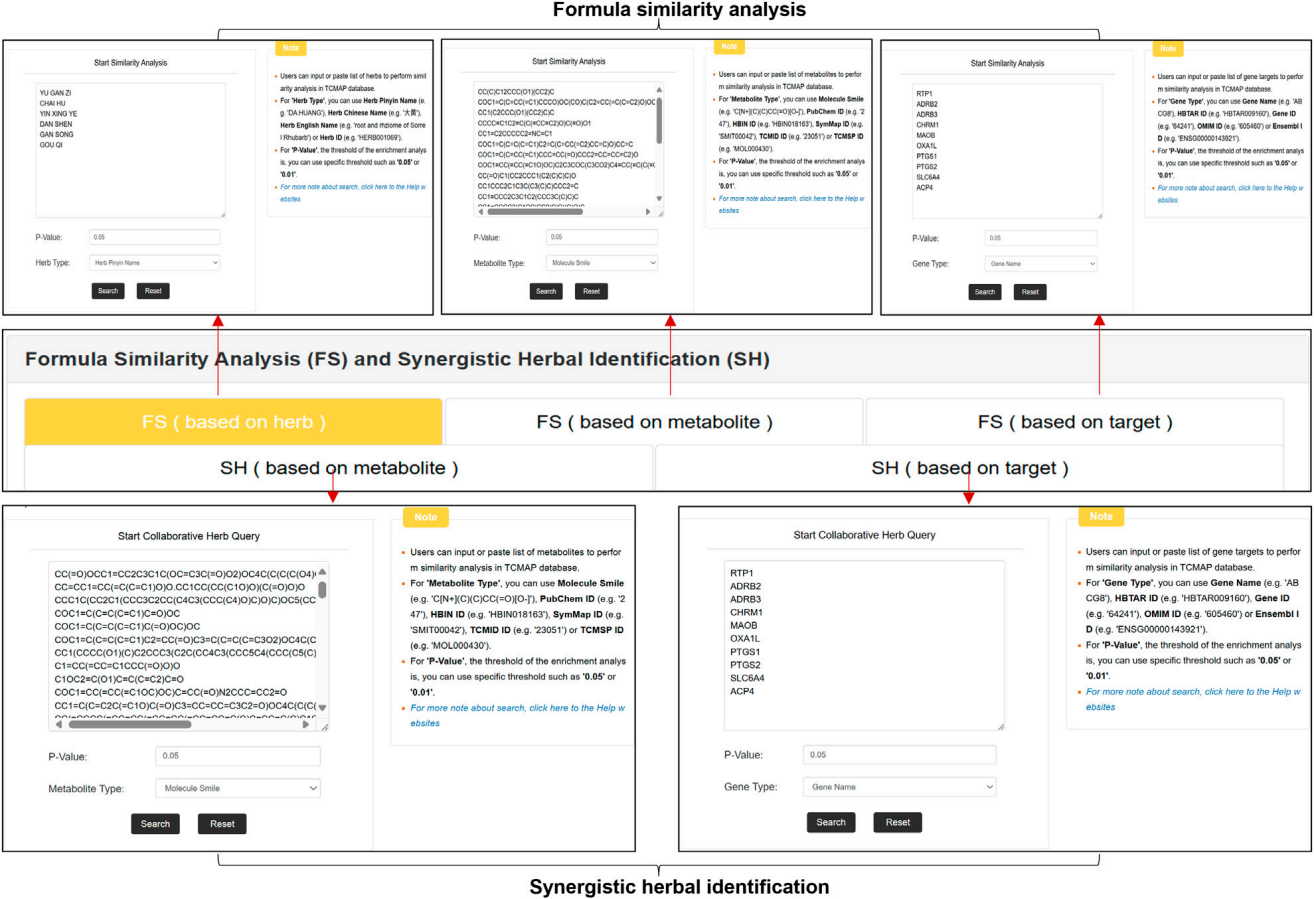
The web interface of the TCMAP webservice. Users can analyze formula similarity based on herbs, metabolites, and gene targets. A list of herbs with synergistic effects can be obtained through metabolites and gene targets.

Similarity analysis helps users explore the compatibility of anti-AP formulas or herbal medicines. Users can enter a pre-prepared list of herbal medicines, metabolites (e.g., HBIN ID), or gene targets (e.g., Gene Name) on the Webserver page. Users can also specify the threshold for the p-value in Fisher’s exact test, where lower p-values indicate a stronger association with anti-AP treatment. The results include a downloadable bubble plot that shows the enrichment level of the tested formulation or herb in anti-AP TCM. Overlap similarity coefficients are used to evaluate the similarity between TCM formulations, with higher coefficients indicating a closer match between samples (Figure 5). Through the above-mentioned formula similarity analysis based on herbs, genes, and metabolites, TCMAP provides users with a more biologically meaningful evaluation of anti-AP formulas, such as metabolite interactions, target pathways, and mechanisms of action.

### Synergistic herbal identification

In recent years, the rapid development of high-throughput sequencing and mass spectrometry technologies has significantly accelerated the discovery of key anti-AP targets and active metabolites (An et al., 2025). Based on this, TCMAP has developed a multidimensional collaborative herbal identification function. Users can input a list of active metabolites or gene targets to systematically analyze the collaborative mechanism of anti-AP herbs from the dual perspectives of chemical structure similarity and biological function correlation (Figure 5).

Users can input active metabolites (such as metabolites in the query prescription) for similarity assessment in the metabolite interaction analysis module. Supported identifiers include chemical structures such as SMILES and public database IDs (PubChem ID, HBIN ID, SymMap ID). The system quantifies the structural similarity of metabolites through the Tanimoto coefficient to obtain a set representing the query metabolites and database metabolites. Users can quickly identify potential collaborative combinations with similar active metabolites by screening significant interactions through Fisher’s exact test (default threshold p ≤ 0.05).

The gene enrichment analysis module allows users to input AP-related genes or gene identifiers (gene name, HBTAR ID, OMIM ID, etc.). The system calculates the degree of enrichment of herbal targets and queries genes using the Jaccard coefficient. Based on the KEGG database to annotate key pathways, this function can recommend herbal combinations targeting key AP pathological mechanisms.

### Using the TCMAP database: case study of webservice

#### Similarity analysis of anti-AP formulations

**FIGURE 6:**
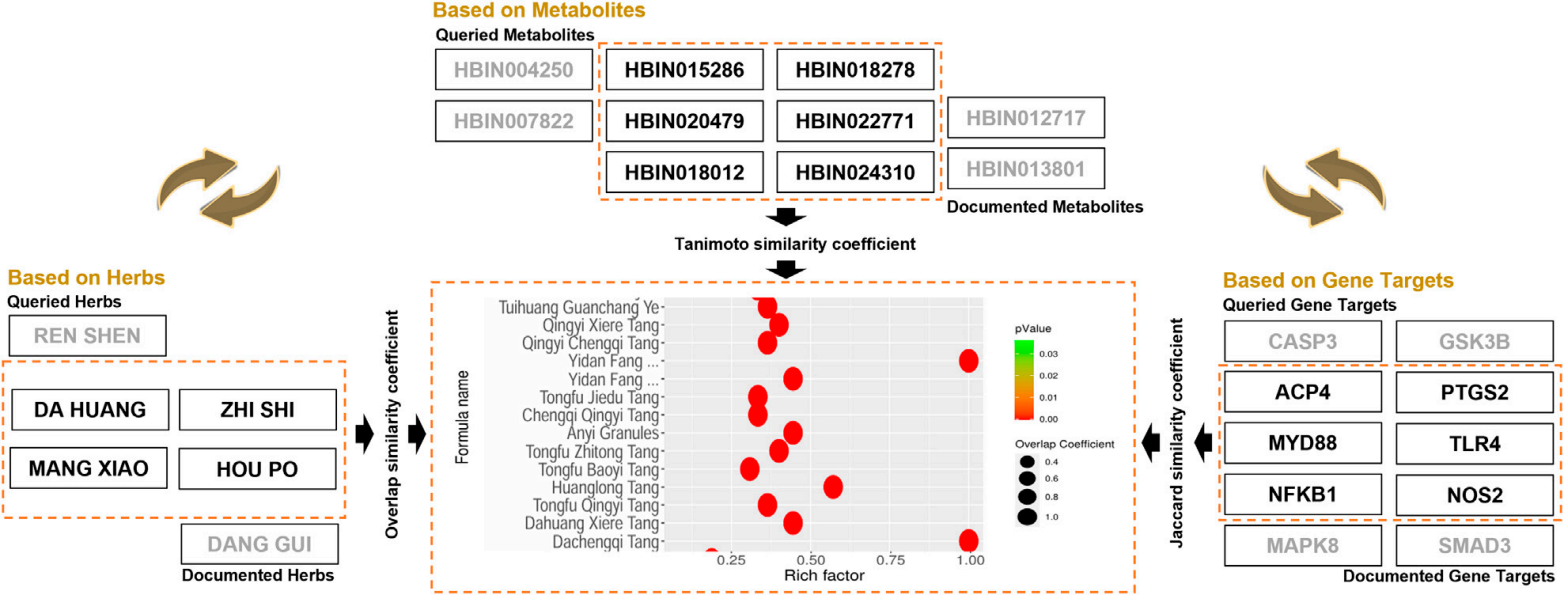
Illustration of the principle and result of formula similarity analysis for herbs, metabolites, and gene targets. By inputting the lists of herbs, active metabolites, and potential gene targets of Dachengqi Decoction, respectively, TCMAP can output anti-AP formulas with similar intrinsic attributes.

Similarity analysis provides innovative research ideas for analyzing and explaining TCM formulas’ material basis and potential mechanisms. Taking the classic anti-AP formula Dachengqi Decoction as an example, its composition, including DA HUANG (*L. Rhubarb*), MANG XIAO (*L. Mirabilite*), ZHI SHI (*L. Immature Bitter Orange*), and HOU PO (*L. Magnolia Bark*), has been verified to have significant curative effects through clinical practice. Based on the multi-dimensional similarity analysis function of TCMAP, users can comprehensively evaluate the clinical applicability of other formulas from three aspects: herbal composition, active metabolites, and target pathways, and explore potential synergistic mechanisms (Figure 6). In the formula analysis module, when the core herbs of Dachengqi Decoction are inputted, TCMAP identifies highly similar formulas such as Dahuang Xiere Decoction, Tongfu Qingyi Decoction, and Huanglong Decoction through the similarity coefficient (*FS*_*herb*_ *= 0.99*). We also note that, compared with Dachengqi Decoction, Huanglong Decoction has newly added DANG GUI (*L. Angelica Sinensis*) and REN SHEN (*L. Ginseng*) to play a tonifying effect. Secondly, by inputting the known active metabolites of Dachengqi Decoction (such as emodin, aloe-emodin, and baicalin), TCMAP screens out highly similar formulas, Yidan Fang (*FS*_*metabolite*_ *= 0.08*). Although the complex metabolites of Yidan Fang against AP have not been fully elucidated, TCMAP analysis suggests that emodin, aloe-emodin, and baicalin may be the key metabolites. Thirdly, by inputting the potential targets of Dachengqi Decoction (ACP4, PTGS2, MYD88, and TLR4), TCMAP enriches Jianwei Qingyi Heji for users. (*FS*_*gene target*_ *= 0.11*). Traditional target analysis requires months for experimental verification. In contrast, the similarity analysis of TCMAP can quickly predict the potential mechanisms of these formulas, which involve regulating pancreatic enzyme activation, inflammatory response, and oxidative stress pathways. Therefore, the similarity analysis of TCMAP accelerates the mechanism analysis of classical prescriptions and provides a data-driven strategy for designing new metabolite formulas.

#### Identification of synergistic anti-AP herb combinations

**FIGURE 7:**
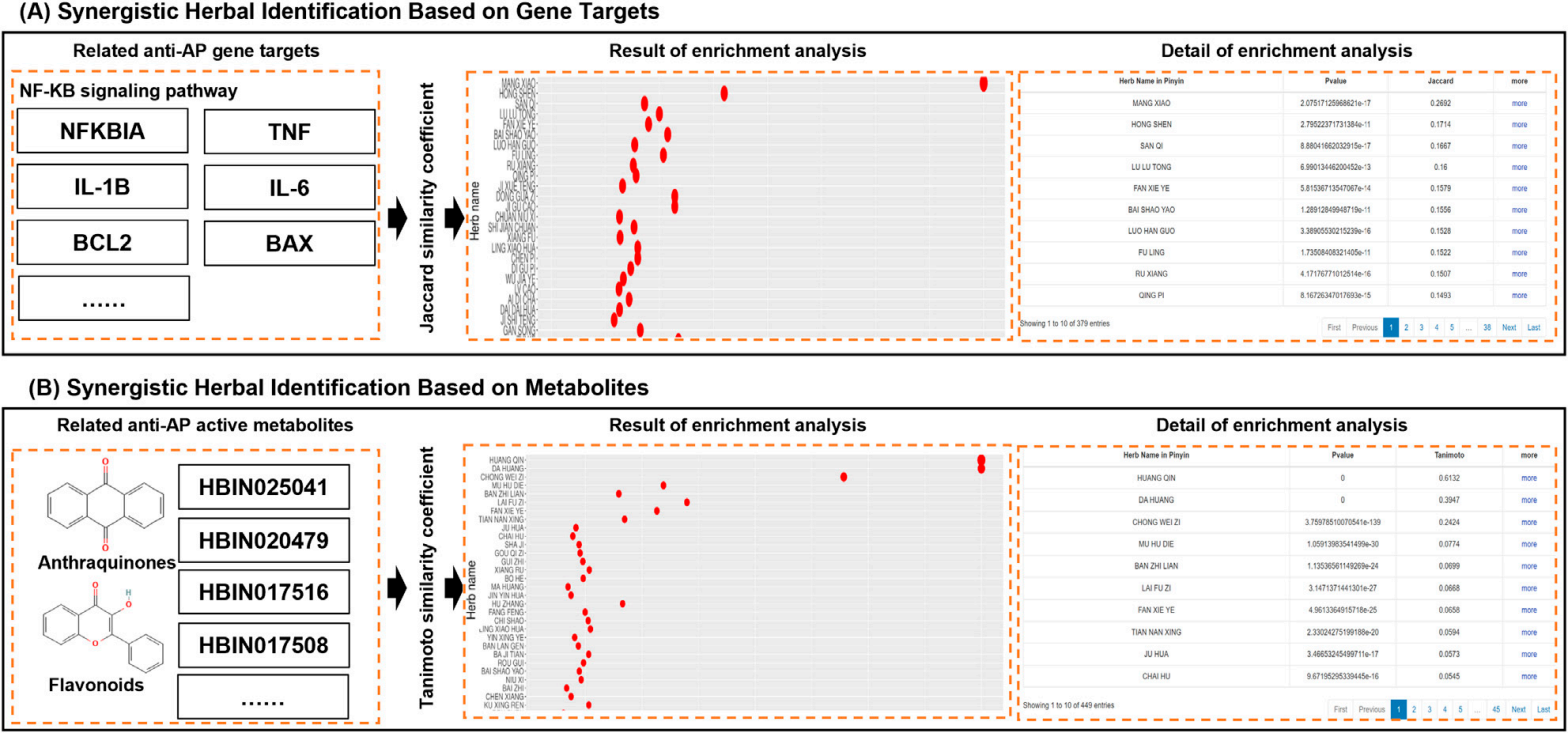
Illustration of the principle and result of synergistic herbal identification for metabolites and gene targets. **(A)** Identifying anti-AP herb combinations targeting NF-κB pathway through the TCMAP database. **(B)** Unveiling synergistic anti-inflammatory herb pairs through metabolite-based analysis using the TCMAP database.

Identifying Anti-AP Herb Combinations Targeting NF-κB Pathway (Figure 7A). AP is characterized by dysregulated inflammatory cascades, with hyperactivation of the NF-κB pathway being a key driver of cytokine storms and clinical mortality. To address this, TCMAP’s gene-based herb similarity analysis module allows users to input NF-κB-associated genes (e.g., NFKBIA, TNF, IL-1B, IL-6, BCL2, BAX) for enrichment analysis. The output ranks herbs by similarity scores, revealing potential candidates such as MANG XIAO, HONG SHEN (*L. Red Ginseng*), SAN QI (*L. Panax notoginseng*), FU LING (*L. Poria*), and CHAI HU (*L. Bupleurum*) (GS _gene_ = 0.27, 0.17, 0.16, 0.15, 0.10). Notably, HONG SHEN and SAN QI, QI-previously unreported for AP treatment, showed significant enrichment for NF-κB pathway inhibition. This discovery highlights TCMAP’s ability to uncover novel therapeutic candidates by integrating molecular targets and clinical hypotheses.

Discovering Synergistic Anti-Inflammatory Herb Pairs via metabolite Analysis (Figure 7B). Anthraquinones (e.g., *L. Emodin, Chrysophanol*) and flavonoids (e.g., *L. Baicalin, Baicalein*) are known for their synergistic anti-inflammatory and antioxidant effects in AP (Hu et al., 2021; Zhao and Zheng, 2023). Using TCMAP’s metabolite-based similarity analysis, users can input compound IDs (e.g., PubChem CID 3220 for Emodin) and set a significance threshold (*P* < 0.05). The study identified Da Huang and Huang Qin (*L. Scutellaria baicalensis*) as a high-similarity pair due to shared anthraquinone-flavonoid synergy (MS _metabolite_ = 0.39 and 0.61). Additionally, Mu Hu Die (*L. Oroxylum indicum*) and Chong Lou (*L. Paris polyphylla*) exhibited structural similarity, suggesting unexplored anti-AP potential (MS _metabolite_ = 0.24 and 0.08). This demonstrates TCMAP’s capacity to guide hypothesis-driven exploration of herbal synergies beyond conventional knowledge.

These case studies demonstrate TCMAP’s unique role in integrating TCM principles with modern biomedical insights. By uncovering novel herb combinations, predicting synergistic pairs, and streamlining formula validation, TCMAP serves as a transformative platform for AP therapeutic discovery. Future updates will enhance clinical and multi-omics data integration.

#### The web interface of the TCMAP network

**FIGURE 8:**
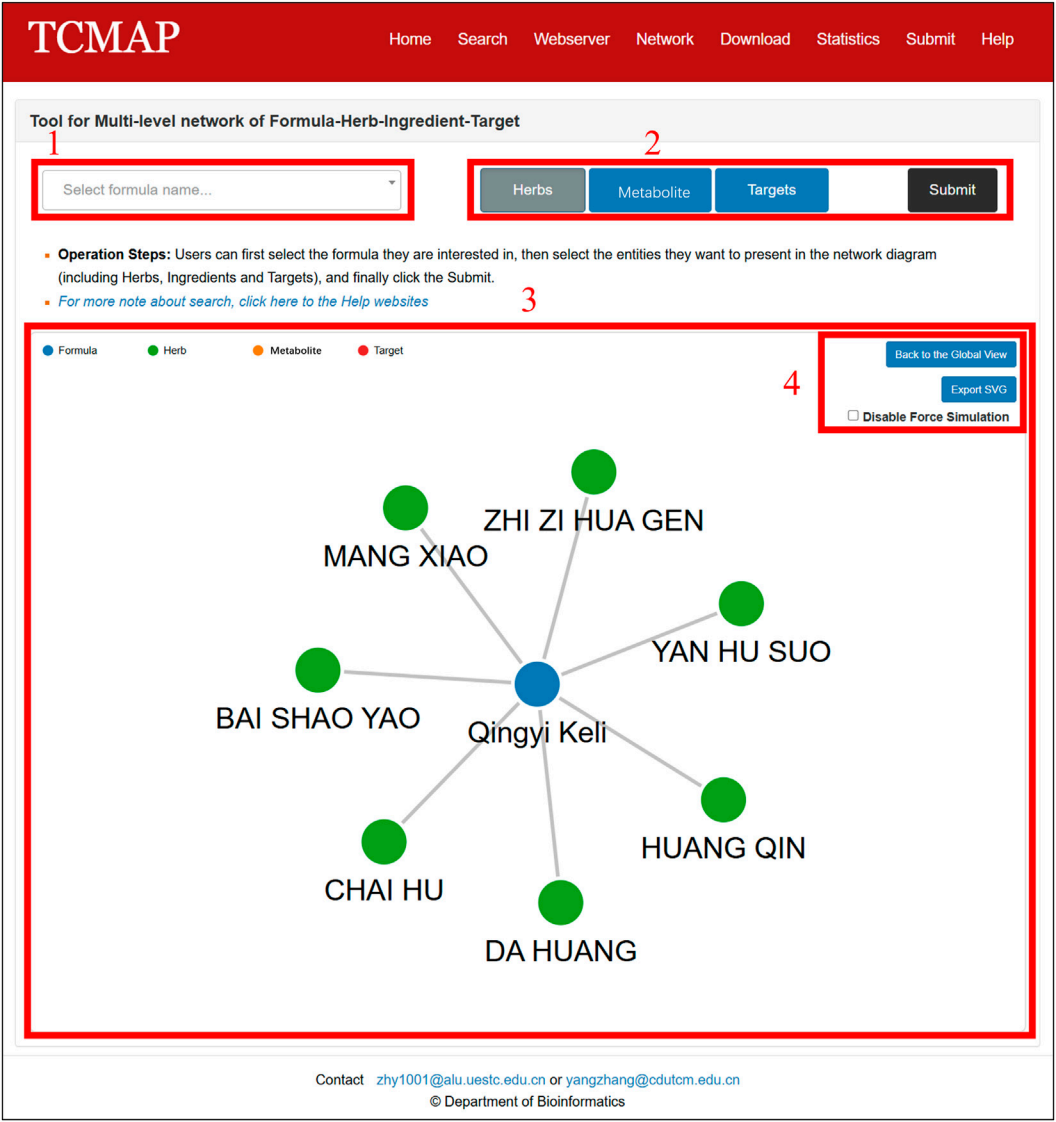
The web interface of the TCMAP network tool. The TCMAP network tool allows users to visualize the multi-level relationships between TCM formulas, herbs, chemical metabolites, and biological targets. Step 1. Formula Selector: A dropdown menu where you choose the herbal formula you want to explore; Step 2. Entity Filters & Submit; Step 3. Network Display; Step 4. View Controls: Options to return to the global overview, export the current graph as SVG.

The Network is a visualization window for analyzing the interaction relationships among formulas, herbs, metabolites, and targets (Figure 8). Users can first select the formula they are interested in, then choose the entities they want to display in the network diagram (including Herbs, metabolites, and Targets), and finally click the Submit button. In the visualization window, users can obtain the herb-metabolite, herb-target, metabolite-target, and herb-metabolite-target graphs of the target anti-AP formula, respectively. In the network diagram, blue nodes represent anti-AP formulas, green nodes represent herbs, orange nodes represent metabolites, and red nodes represent targets. Through network analysis, users can quickly and comprehensively understand the key metabolites and key targets of anti-AP formulas.

#### Using the TCMAP database: a case study of network analysis

**FIGURE 9:**
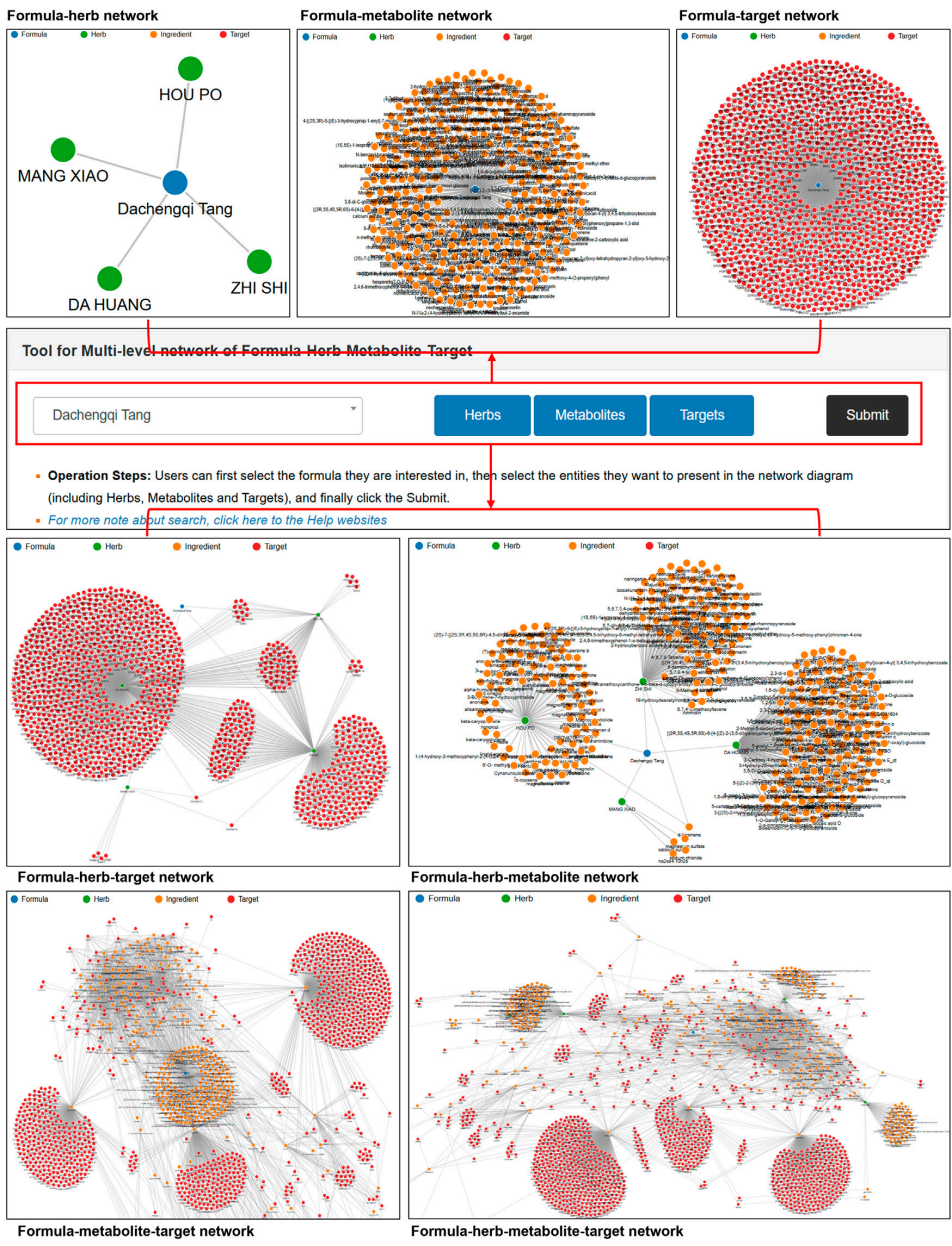
Illustration of the result of network analysis for Dachengqi Tang. y visualizing the multi - dimensional relationships among the herbs, metabolites, and targets of anti - AP formulas, TCMAP offers users a convenient approach for network pharmacology analysis.

Taking Dachengqi Tang as an example, the network analysis function of TCMAP can systematically analyze its multi-level action network (formula-herb-metabolite-target) against AP. Users can input Dachengqi Tang through the drop-down menu, sequentially select the herb, metabolite, and target levels, and then submit. After that, the platform automatically generates interactive network diagrams in different dimensions (Figure 9). In the formula-herb-target association network, the core herbs DA HUANG, ZHI SHI, and HOU PO cooperatively regulate targets such as NOS2, IL-6, and GSK3β, exerting anti-AP effects by inhibiting inflammatory responses and oxidative stress. Further analysis of the formula-herb-metabolite network reveals that d-limonene is a common metabolite of ZHI SHI and HOU PO, while DA HUANG exhibits the broadest spectrum of active metabolites (such as rhein and aloe-emodin), covering most of the key targets. In the metabolite-target interaction network, the ZHI SHI metabolite citric acid shares three targets (GNAI1, GNAI3, AANAT) with serotonin. Citric acid and eugenol jointly target 17 genes (MME, CTSD, BMP6, RRM2B, PRDX3, etc.), and serotonin and eugenol overlap in regulating nine targets (CXCR4, CCR2, PPBP, KCNJ2, etc.). These interaction relationships reveal the potential of metabolites to synergistically regulate AP-related pathogenic targets. Through visualizing the multi-dimensional associations within the compound formula, the network analysis of TCMAP accelerates the screening of key metabolites and core targets of anti-AP formulas, providing an intuitive data-driven tool for mechanism analysis and drug development.

## Discussion

In recent decades, TCM databases have rapidly advanced, significantly enhancing the storage and analysis of complex pharmacological data (Xu et al., 2019; Zhao et al., 2023; Elizarraras et al., 2024). Although several comprehensive TCM databases have successfully integrated extensive data on herbs, metabolites, TCM syndromes, and target genes, users still encounter difficulties understanding the combinations and developmental value of different herbs (Zeeshan et al., 2020; Kong et al., 2024). This issue stems from TCM prescriptions being frequently adjusted according to specific disease conditions. Existing comprehensive TCM databases that rely on herb-metabolite-target networks do not adequately capture the particular patterns and potential mechanisms of TCM formulations for various diseases. Therefore, creating a robust and independent database dedicated to complex and epidemic diseases with proven TCM treatment advantages—such as COVID-19 (Yu et al., 2024), malaria (van der Kooy and Sullivan, 2013), AP (Qiong et al., 2005), and fatty liver disease (Zhou et al., 2022; Gong et al., 2024)—would more effectively facilitate the development of new therapeutic strategies and the elucidation of underlying mechanisms.

TCM provides multi-target and multi-organ therapeutic benefits in preventing and treating AP and its associated complications, with its efficacy being widely acknowledged domestically and internationally (Qiong et al., 2005; Zheng et al., 2015; Zhou et al., 2016). To expedite the development of new therapies for AP and its related complications, we have developed a database that consolidates foundational and clinical research evidence in TCM for AP. This database is designed to optimize the integration of research findings in this area, facilitating advancements in therapeutic strategies.

Compared with existing TCM databases, TCMAP is more focused on integrating the research progress of formulas for treating AP and its complications, including potential mechanisms, serum metabolites, and clinical trial evidence (Table 1). Although ETCM2 also contains TCM formulas, it emphasizes the classification of efficacy (heat-clearing, tonic, and exterior-releasing herbs) without specific disease information (Zhang et al., 2023). The TCMSP and HERB databases provide comprehensive information on herbs and metabolites but lack TCM formula data (Ru et al., 2014). The SymMap database includes extensive disease and target data from the perspective of syndrome types but does not provide specific descriptions of AP-related treatments and mechanisms (Wu et al., 2019). Meanwhile, to more intuitively show users the situation of the entered formulas, TCMAP also provides a webservice through similarity analysis of anti-AP formulas based on herbs, metabolites, and genes. It can comprehensively evaluate the clinical applicability of existing formulas at the level of intrinsic attributes. This is not available in existing TCM databases. Furthermore, TCMAP also provides network analysis of anti-AP formulations based on herbs, metabolites, and targets. Based on the results of the network analysis, it further explains the key metabolites and potential targets in the anti-AP formulations to users. Therefore, the irreplaceability of TCMAP in AP research fills the gaps in existing databases.

**TABLE 1:** Comparison of representative TCM databases.

Category	TCMAP	HERB	ETCM2	TCMSP	SymMap	TCM2COVID
Disease Focus	AP	General	General	General	General	COVID-19
TCM Formulae	200	N/A	3,959	N/A	N/A	300
Herbs	449	7263	402	499	698	320
Natural metabolites	58	49,258	7248	29,384	25,975	90
Serum metabolites	13	N/A	N/A	N/A	N/A	N/A
Disease-Target Links	AP-specific pathways	28,212	4323	837	14,086	COVID19-specific pathways
Clinical Evidence	Linked to trials	No	No	No	No	No
Functional Tools	AP-Specific similarity analysis	N/A	Systematic analysis	N/A	N/A	Specific similarity analysis
Unique Features	Serum data integration Clinical linkage	Broad metabolite coverage	Formula classification	metabolite-target networks	Symptom mapping	“Formula-Herb-Disease multi-level Knowledge Graph Network” for COVID-19

The TCMAP database is a meticulously curated repository for TCM treatments of AP. Its innovations are as follows: (i) TCMAP compiles authentic experimental data on AP and organizes high-quality TCM resources for researchers. We implemented a two-step verification process to ensure the reliability and accuracy of the extracted data. After each data extraction, the dataset undergoes cross-validation by two independent reviewers. They meticulously compare the extracted data against the sources to identify discrepancies or errors. Users can access detailed information on anti-AP herbal formulas, including dosage form, administration, classification, herb metabolites, application range, potential mechanisms, and China Clinical Trial registry details. (ii) TCMAP emphasizes the systemic information of blood-active metabolites in formulas, offering valuable data for TCM network analysis. Recent studies have highlighted the serum pharmacochemical characteristics of anti-AP formulas, which facilitate more precise metabolite-disease-target network analysis compared to single herb data. (iii) TCMAP provides free similarity analysis and data network visualization for anti-AP formulas or herbs. The web interface lets users efficiently query and download enrichment data for tested herbal formulas. In summary, TCMAP aims to integrate TCM data related to treating AP, surpassing traditional TCM databases.

Furthermore, the TCMAP database has several limitations. Firstly, there is the issue of standardizing herb names. The same herb may have aliases in different regions or historical documents. Although we rely on authoritative pharmacopoeias for mapping, a small number of entries cannot be automatically matched. Secondly, the clinical evidence is fragmented. Some formulas only have animal experiment data and lack support from RCTs. In the future, we need to strengthen cooperation with clinical registration platforms. Thirdly, due to the incomplete nature of the sources, the database lacks information on the processing methods of the herbs used in the recorded formulations. In response to the above-mentioned technical deficiencies, we will continuously collect feedback data from actual users, including the results of user experience surveys and satisfaction surveys, to constantly promote the expansion and improvement of the TCMAP database.

## Conclusion

The TCMAP database provides users with an intuitive platform that enables easy browsing, searching, visualizing, and downloading anti-AP TCM research data and conducting similarity and enrichment analyses of critical prescriptions. However, clinical research data on anti-AP herbal medicine remains relatively scarce during the data integration process. Future efforts are needed to conduct large-scale clinical trials to provide higher-quality evidence for TCM treatments against AP. In summary, the TCMAP database comprehensively consolidates publicly available research data on TCM for AP, which will facilitate the elucidation of the potential mechanisms of TCM in treating AP and the discovery of new herbal formulas with anti-AP properties.

## Data Availability

The original contributions presented in the study are included in the article/Supplementary Material, further inquiries can be directed to the corresponding authors.

## Supplementary material

SUPPLEMENTARY TABLE S1 The detail information of formulas in TCMAP.

SUPPLEMENTARY TABLE S2 The detail information of natural metabolites in TCMAP.

SUPPLEMENTARY TABLE S3 The detail information of herbs in TCMAP.

SUPPLEMENTARY TABLE S4 The detail information on the top 10 significant pathway results of KEGG enrichment analysis for formulas.

## Abbreviations

TCM, Traditional Chinese medicine; AP, acute pancreatitis; CT, Computed Tomography; CTSB, lysosomal cathepsin B; ORAI1, calcium channel protein1; NF-κB, nuclear factor-κB; ROS, reactive oxygen species; LC-MS/MS, liquid chromatography-tandem mass spectrometry; UA, Urolithin A; EA, Ellagic acid; SAP, Severe acute pancreatitis; CNKI, China National Knowledge Infrastructure; RCTs, Randomized controlled trials; FS, formula similarity; MS, metabolite similarity; GS, gene similarity; HTML, HyperText Markup Language; CSS, Cascading Style Sheets; AJAX, Asynchronous JavaScript and XML; PHP, Hypertext Preprocessor.
